# Supplementary material for: Effect of Deacidification Treatment on the Flavor Quality of Zaosu Pear–Kiwifruit Wine
Source: Foods. 2022 Jul 7;11(14):2007. doi: 10.3390/foods11142007 (PMC9324503; doi:10.3390/foods11142007)
Supplement: Supplementary file 1 [file foods-11-02007-s001.zip › foods-1794561-supplementary.pdf]

Table S1 Standard curve equation of seven organic acids.

Compounds	Retention time (min)	Regression equation	correlation coefficient (R^2)
Oxalic acid	5.969	y=878.829x	0.9994
L-malic acid	6.551	y=696.775x	0.9995
Citric acid	11.507	y=747.495x	0.9991
Succinic acid	13.267	y=520.039x	0.9997
Quinic acid	5.312	y=1726.84x	0.9993
Tartaric acid	5.900	y=221.156x	0.9991
Lactic acid	7.986	y=407.078x	0.9996

Table S2 Standard curve equation of seven polyphenols.

Compounds	Retention time (min)	Regression equation	correlation coefficient (R^2)
Protocatechuic acid	5.430	y=24019.7x-16240.1	0.9992
Chlorogenic acid	10.464	y=11465.8x-12492.7	0.9993
Gallic acid	24.068	y=11444.4x-13123.8	0.9993
Epicatechin	28.066	y=4707.05x-5854.57	0.9993
Catechin	4.321	y=1839.95x	0.9991
Caffeic acid	19.159	y=7519.72x	0.9991
Phloretin	22.877	y=1646.81x	0.9995

Table S3. Organic acid concentration of pear-kiwifruit juice and corresponding wines with and without deacidification treatment.

Compounds	Organic acid concentration (g/L)			
	Mixed juice [a]	AF wine [b]	MLF wine [c]	CD wine[d]
Oxalic acid	0.78±0.006^{a}	0.76±0.007^{b}	0.66±0.002^{c}	0.44±0.003^{d}
L-malic acid	1.97±0.015^{a}	1.80±0.012^{b}	0.04±0.001^{c}	1.70±0.01^{b}
Citric acid	0.59±0.002^{d}	0.80±0.005^{a}	0.73±0.001^{c}	0.75±0.006^{b}
Succinic acid	0.10±0.001^{b}	0.11±0.002^{a}	0.06±0.001^{d}	0.08±0.001^{c}
Quinic acid	1.53±0.009^{a}	1.13±0.005^{b}	0.93±0.004^{d}	0.99±0.006^{c}
Tartaric acid	1.43±0.009^{a}	1.38±0.003^{b}	0.99±0.012^{c}	0.39±0.004^{d}
Lactic acid	0.22±0.006^{c}	0.64±0.005^{b}	1.23±0.004^{a}	ND

All values are reported as the mean (± SD) of three experiments. ND: not detected.

Values in the same row with different letters indicate statistically different by Tukey's test ($P < 0.05$).

[a] Mixed juice: pear and kiwifruit juice at the blend ratio of 60:40.

[b] AF wine: pear-kiwifruit juice co-inoculated with *Saccharomyces cerevisiae* ES488 and *Metschnikowia. pulcherrima* 346.

[c] MLF wine: AF wine inoculated with *Oenococcus oeni* strain GF-2.

[d] CD wine: Chemically deacidified wine by 1.0 g/L Na_2CO_3, 1.0 g/L K_2CO_3 and 5.0 g/L $KHC_4H_4O_6$.

Table S4 Polyphenol concentration of pear-kiwifruit juice and corresponding wines with and without deacidification treatment.

Compounds	Polyphenol concentration (μg/mL)			
	Mixed juice [a]	AF wine [b]	MLF wine [c]	CD wine[d]
Protocatechuic acid	3.52±0.02[b]	3.58±0.01[ab]	3.62±0.05[a]	ND
Chlorogenic acid	8.26±0.03[c]	10.41±0.17[b]	11.06±0.09[a]	5.80±0.01[d]
Gallic acid	6.17±0.04[b]	6.05±0.01[c]	6.49±0.08[a]	5.97±0.04[d]
Epicatechin	6.33±0.01[c]	7.85±0.13[a]	6.60±0.03[b]	6.39±0.01[c]
Catechin	10.17±0.14[c]	12.70±0.13[a]	11.11±0.15[b]	5.38±0.08[d]
Caffeic acid	1.38±0.03[b]	1.21±0.02[c]	1.52±0.07[a]	0.68±0.02[d]
Phloretin	3.30±0.06[c]	5.77±0.09[a]	4.26±0.09[b]	2.02±0.05[d]

All values are reported as the mean (± SD) of three experiments. ND: not detected.

Values in the same row with different letters indicate statistically different by Tukey's test ($P < 0.05$).

[a] Mixed juice: pear and kiwifruit juice at the blend ratio of 60:40.

[b] AF wine: pear-kiwifruit juice co-inoculated with *Saccharomyces cerevisiae* ES488 and *Metschnikowia. pulcherrima* 346.

[c] MLF wine: AF wine inoculated with *Oenococcus oeni* strain GF-1.

[d] CD wine: Chemically deacidified wine by 1.0 g/L Na_2CO_3, 1.0 g/L K_2CO_3 and 5.0 g/L $KHC_4H_4O_6$.

Table S5 Volatile aroma compounds in pear-kiwifruit juice and corresponding alcohol fermentation or deacidification wines.

Number	Compouds	Aroma compounds concentration (μg/L)			
		Mixed juice [a]	AF wine [b]	MLF wine [c]	CD wine [d]
A1	Ethyl acetate	239±11[b]	227±4[c]	278±12[a]	100±10[d]
A2	Ethyl butanoate	15.4±4.7[c]	69.6±4.6[a]	66.2±8.3[a]	24.3±0.9[b]
A3	n-Ethyl propanoate	4.9±2.3[a]	3.3±1.6[b]	3.8±0.3[b]	ND
A4	n-butyl acrylate	0.24±0.01[a]	ND	ND	ND
A5	Phenethyl acetate	138±16[b]	198±60[a]	216±5[a]	110±11[b]
A6	Isobutyl acetate	ND	7.5±0.3[a]	12.5±7.8[a]	ND
A7	Hexyl acetate	503±24[a]	425±19[c]	477±11[b]	210±14[d]
A8	Isoamyl acetate	483±31[b]	1231±98[a]	1305±166[a]	349±18[c]
A9	Ethyl hexanoate	1037±98[a]	1014±131[a]	1138±88[a]	420±13[b]
A10	3-Methylbutyl hexanoate	32.6±13.7[a]	13.4±1.9[b]	10.1±1.7[b]	ND
A11	Methyl hexoate	22.2±5.2[a]	13.7±0.4[b]	ND	ND
A12	(3Z)-3-Hexen-1-yl acetate	66.6±10.8[a]	21.6±4.6[c]	48.7±0.6[b]	ND
A13	Ethyl heptanoate	ND	5.1±0.7[b]	11.5±0.2[a]	ND
A14	Heptyl acetate	3.6±0.3[b]	7.6±0.7[a]	8.4±0.4[a]	ND
A15	2-Methylpropyl octanoate	7.3±1.7[c]	14.5±0.7[a]	11.3±1.0[b]	ND
A16	Ethyl nonanoate	12.0±5.5[b]	22.0±1.8[a]	24.9±2.4[a]	ND
A17	Methyl octylate	35.8±4.9[a]	37.8±6.7[a]	41.7±1.1[a]	ND
A18	Ethyl caprylate	2899±116[a]	3129±485[a]	3458±498[a]	1979±47[a]
A19	Hexyl octanoate	3.6±0.5[b]	2.9±1.0[a]	ND	ND
A20	3-Methylbutyl octanoate	42.6±5.2[c]	76.7±2.9[a]	61.6±3.4[b]	42.6±2.1[c]
A21	Methyl caprate	ND	12.5±1.7[a]	7.3±0.3[b]	ND
A22	Ethyl caprate	874±79 [c]	1485±122[b]	2188±150[a]	341±17[d]
A23	Isoamyl decanoate	ND	36.40±1.17[a]	38.62±1.38[a]	ND
A24	Isobutyl decanoate	ND	25.42±7.46[a]	17.88±7.99[a]	ND

A25	Diethyl succinate	3.2±0.2^{b}	4.6±0.8^{b}	552±43^{a}	2.0±0.7^{b}
A26	Ethyl laurate	320±18^{b}	716±21^{a}	708±5^{a}	304±6^{b}
A27	Ethyl tetradecanoate	1.1±0.3^{b}	6.9±0.6^{a}	8.6±1.2^{a}	ND
A28	Ethyl hex-3-enoate	4.4±0.9^{a}	1.3±0.1^{b}	1.5±0.2^{b}	ND
A29	7-Octenoic acid ethyl ester	ND	2.0±0.1^{a}	2.7±0.6^{a}	ND
A30	Ethyl (*E*)-2-hexenoate	8.5±2.2^{a}	2.4±0.4^{b}	2.1±0.2^{b}	0.4±0.1^{b}
A31	Citronellol acetate	6.2±1.4^{a}	4.3±0.4^{a}	ND	ND
A32	methyl (2*E*,4*Z*)-deca-2,4-	2.8±0.3^{b}	1.3±0.1^{b}	4.2±0.3^{a}	ND
A33	ethyl (2*E*,4*Z*)-deca-2,4-	718±18^{b}	730±27^{b}	879±41^{a}	6.3±1.1^{c}
A34	Ethyl undecylenate	7.4±1.0^{a}	7.8±1.0^{a}	7.3±0.6^{a}	ND
A35	Propyl octanoate	5.5±1.4^{a}	ND	ND	ND
A36	Ethyl palmitate	0.59±0.04^{c}	2.4±0.1^{a}	1.1±0.5^{b}	ND
A37	Ethyl lactate	ND	ND	1.9±0.1^{a}	ND
A38	Methyl salicylate	2.4±0.1^{b}	ND	4.9±0.4^{a}	ND
	**Total esters**	**7500±237^{c}**	**9558±1001^{b}**	**11598±1057^{a}**	**3889±143^{d}**
B1	Isobutanol	35.6±12.8^{b}	103.4±8.8^{a}	102.7±2.5^{a}	33.0±1.7^{b}
B2	Pentanol	1029±142^{b}	3264±70^{a}	3452±121^{a}	1087±48^{b}
B3	1-Octanol	10.7±2.1^{b}	20.7±4.8^{b}	39.3±1.4^{a}	14.4±0.3^{b}
B4	1-Hexanol	650±42^{a}	480±35^{a}	527±17^{a}	249±16^{b}
B5	1-Heptanol	50.1±5.2^{a}	51.0±4.1^{a}	54.2±1.5^{a}	42.9±1.8^{b}
B6	Decan-1-ol	ND	9.5±2.2^{a}	9.5±2.9^{a}	5.8±0.4^{a}
B7	Phenylethyl alcohol	528±64^{d}	1398±48^{b}	1780±85^{a}	724±41^{c}
B8	Benzyl alcohol	ND	ND	10.0±0.4^{a}	ND
B9	3-Methylpentanol	ND	4.8±0.7^{a}	4.4±0.3^{a}	ND
B10	(*E*)-3-Hexen-1-ol	15.3±2.6^{c}	54.4±3.6^{a}	32.1±2.3^{b}	ND
B11	(*Z*)-4-Hepten-1-ol	ND	2.9±0.1^{a}	3.3±0.6^{a}	ND
B12	2-Ethyl-1-hexanol	6.7±1.3^{a}	1.4±0.1^{d}	3.2±0.2^{c}	4.4±0.4^{b}
B13	2, 3-Butanediol	ND	2.7±0.5^{b}	20.4±3.3^{a}	0.60±0.06^{b}
B14	Isopulegol	3.3±0.5^{b}	3.4±0.3^{b}	7.1±0.1^{a}	2.1±0.1^{c}
B15	(*E*)-2-Decen-1-ol	ND	ND	1.5±0.3^{a}	ND
B16	5-Methylheptan-3-ol	ND	ND	7.2±0.2^{a}	ND
B17	(*Z*)-3-Hexen-1-ol	13.6±2.9^{b}	11.9±0.3^{b}	71.1±3.6^{a}	15.9±0.9^{b}
B18	1-Nonanol	0.93±0.03^{a}	ND	ND	0.91±0.05^{a}
	**Total alcohols**	**2343±168^{c}**	**5408±178^{b}**	**6125±238^{a}**	**2180±112^{c}**
C1	Isobutyric acid	4.2±1.0^{b}	9.8±0.7^{a}	8.1±0.4^{a}	4.9±0.7^{b}
C2	Octanoic acid	326±20^{a}	276±17^{b}	341±5^{a}	70.9±5.3^{c}
C3	Heptanoic acid	1.3±0.5^{a}	1.3±0.3^{a}	1.4±0.2^{a}	ND
C4	9-Decenoic acid	ND	ND	2.3±0.7^{a}	ND
C5	(*Z*)-5-dodecenoic acid	4.5±1.4^{a}	3.7±0.1^{a}	ND	ND
C6	2-Methylbutyric acid	16.2±2.9^{b}	45.9±3.6^{a}	ND	6.2±0.4^{c}
C7	3-Hydroxycinnamic acid	ND	ND	1.2±0.1^{a}	ND
C8	nonanoic acid	1.9±0.1^{a}	ND	ND	ND
	**Total volatile fatty acids**	**354±24^{a}**	**337±22^{a}**	**354±6^{a}**	**82±7^{b}**
D1	α-Terpineol	3.8±1.1^{b}	4.3±0.3^{b}	15.1±0.6^{a}	1.9±0.2^{c}
D2	Citronellol	8.9±2.4^{c}	14.0±0.5^{b}	18.7±0.7^{a}	9.8±0.5^{c}
D3	Nerol	2.4±0.1^{b}	ND	7.0±0.2^{a}	2.4±0.6^{b}
D4	β-damascenone	3.1±1.2^{a}	2.4±0.8^{a}	1.9±0.6^{a}	ND
D5	Linalool	16.2±3.5^{b}	15.4±1.5^{b}	33.3±1.8^{a}	10.6±0.6^{c}
D6	Terpinen-4-ol	24.7±4.2^{a}	21.0±1.7^{a}	ND	ND
D7	Lauryl alcohol	ND	ND	4.7±0.9^{a}	ND
	**Total terpenoids**	**59±13^{b}**	**57±5^{b}**	**81±8^{a}**	**25±4^{c}**
E1	Nonanal	0.87±0.05^{a}	ND	ND	ND
E2	Decanal	4.1±1.3^{a}	ND	ND	ND
E3	Sulcatone	5.1±1.7^{a}	6.2±0.2^{a}	ND	2.4±0.2^{b}

E4	Methyl eugenol	22.8±3.7[a]	25.7±1.8[a]	24.7±1.2[a]	11.3±0.3[b]
E5	Eugenol	3.0±0.6[b]	2.5±0.2[b]	18.6±1.9[a]	3.3±0.1[b]
E6	Styrene	ND	ND	15.4±0.7[a]	ND
	**Total other compounds**	**36±8[ab]**	**34±24[ab]**	**59±21[a]**	**17±3[b]**

All values are reported as the mean (± SD) of three experiments. ND: not detected.

Values in the same row with different letters indicate statistically different by Tukey's test ($P < 0.05$).

[a] Mixed juice: pear and kiwifruit juice at the blend ratio of 60:40.

[b] AF wine: pear-kiwifruit juice co-inoculated with *Saccharomyces cerevisiae* ES488 and *Metschnikowia. pulcherrima* 346.

[c] MLF wine: AF wine inoculated with *Oenococcus oeni* strain GF-1.

[d] CD wine: Chemically deacidified wine by 1.0 g/L Na_2CO_3, 1.0 g/L K_2CO_3 and 5.0 g/L $KHC_4H_4O_6$.

Table S6 Sensory descriptors used in the CATA test and their frequencies (n=36) presented in the order of most often selected attributes.

CATA attribute	AF wine [a]	MLF wine [b]	CD wine [c]	*P*-value [d]
Fruity	20	28	5	0.050
perry-like	10	20	3	<0.001
Fermented	24	22	22	0.450
Acidic	24	12	6	0.010
Acidic/fresh	5	23	16	<0.001
Astringent	19	11	13	0.640
Sweet	5	7	8	0.720
Bitter	3	2	26	0.013
Floral	12	21	6	<0.001
Cooked pear	18	27	7	<0.001
Fresh pear	9	16	5	0.020
Alcoholic	10	10	9	0.760
Mild	4	9	7	0.580
Tropical fruit	1	2	1	0.780
Yeasty	11	8	6	0.610
Honey	5	6	3	0.660
Diverse	10	25	8	<0.001
Strong	6	3	5	0.470
Earthy	3	1	3	0.700
Citrus	3	3	2	0.730
Simple	12	1	9	0.017
Spicy	6	1	2	0.240
Sharp	11	2	4	0.063
Chemical	1	0	2	0.580
Grassy	7	7	6	0.720

[a] AF wine: pear-kiwifruit juice co-inoculated with *Saccharomyces cerevisiae* ES488 and *Metschnikowia. pulcherrima* 346.

[b] MLF wine: AF wine inoculated with *Oenococcus oeni* strain GF-1.

[c] CD wine: Chemically deacidified wine by 1.0 g/L Na_2CO_3, 1.0 g/L K_2CO_3 and 5.0 g/L $KHC_4H_4O_6$.

[d] Based on Cochran's Q-test ($P<0.05$).
